# Aflatoxin Contamination in Food and Body Fluids in Relation to Malnutrition and Cancer Status in Cameroon

**DOI:** 10.3390/ijerph7010178

**Published:** 2010-01-18

**Authors:** Angele N. Tchana, Paul F. Moundipa, Félicité M. Tchouanguep

**Affiliations:** Laboratory of Molecular Toxicology and Pharmacology, Department of Biochemistry, University of Yaoundé I, Cameroon; E-Mail: ftchouanguep@yahoo.fr

**Keywords:** aflatoxins, egg, milk, malnutrition, hepatitis B virus, cancer, Cameroon

## Abstract

Aflatoxins are food contaminants usually associated with hepatitis, immunodepression, impairment of fertility and cancer. The present work was to determine the presence of aflatoxins in eggs, milk, urine, and blood samples that were collected from various sources and periods; and hepatitis B virus antigen in blood samples. Aflatoxin was found in eggs (45.2%), cow raw milk (15.9%), breast milk (4.8%), urine from kwashiorkor and marasmic kwashiorkor children (45.5%), and sera from primary liver cancer patients (63.9%); HbsAg was also detected in 69.4% of the serum samples, but there was no association between both factors. Both AF and hepatitis B virus seem to be risk factors that could increase the incidence and prevalence rates of malnutrition and cancer in Cameroon.

## 1. Introduction

Aflatoxins (AFs) are secondary metabolites of the fungi *Apergillus flavus* and *Aspergillus parasiticus*. These moulds are common contaminants of foodstuff, particularly in the tropical regions [1]. Many studies have linked aflatoxin contamination of food with some toxic effects such as liver cancer and immunosupression in various animals and humans [2,3]. Aflatoxin B1, the most potent one, is metabolized into a variety of hydroxylated derivatives (aflatoxin P_1_, M_1_, B_2a_, aflotoxicol) which are less toxic than the parent compound [4], although their presence in food is still a threat to human health [5]. Aflatoxin B_1_ has been detected in human foodstuffs in some African countries [6] and correlated with a high incidence of primary liver cancer. In Cameroon, some of our early reports [7–9] have shown the presence of AFB_1_ and some of its metabolites in foodstuff and animal feed at levels higher than recommended by the guidelines issued by international regulatory committees [10]. In spite of these studies, the relation between aflatoxin contamination in body fluids and states of some diseases such as cancer and malnutrition is not very obvious because of insufficiency of statistical data in our country. This contamination, in addition to infection by some viruses such as hepatitis B or HIV [11,12], could have serious effects on the quality of health in Cameroon. This study was therefore designed to determine the levels of aflatoxins in foods which are recommended as high protein sources for children, like eggs and milk, and the blood and urine of patients suffering from malnutrition and cancer.

## 2. Material and Methods

### 2.1. Sample Collection

All the samples were collected during a four-year survey (1991–1995) from various climatic regions in Cameroon [13] and during various seasons. Egg samples were collected during the rainy season from randomly chosen poultry farms; eggs from the same poultry were pooled together to make one egg sample for extraction. Cow milk was also collected, either from a market or from a herd. Milk collected from different cattle of a herd was pooled together to make a single sample. All samples were kept at −20 °C until analysis. Maternal milk was collected at three different hospitals in Yaounde from mothers who were interviewed and gave their informed consent to participate in the study. The milk was aseptically taken using a sucker and stored at −20 °C until use; information related to delivery, food habits and social rank was collected from the women.

A 24-H urine sample [14] was collected from each of 42 children suffering from kwashiorkor or marasmic kwashiorkor admitted at the University Teaching Hospital in Yaounde, and from each of 36 healthy children within the same age range and area of residence as the patients, and whose parents gave their informed consent. None of the 78 children had any parasitic co-infections, as determined by stool and blood examinations.

Blood samples were also collected from cancer patients admitted in two reference hospitals in Yaounde during a four-year period, following their informed consent. Serum samples were prepared from the blood and kept at −20 °C for aflatoxin and HbsAg detection. Ethical clearance for the study was obtained from the National Ethical Committee at the Ministry of Public Health, Yaounde, Cameroon.

### 2.2. Chemical Analysis

*Aflatoxin extraction and purification*. Aflatoxins were extracted and purified using different methods, depending on the nature of the sample. Egg samples (100 g) were mixed with 42 mL of 40% (w/v) NaCl and then extracted with acetone according to the AOAC method as modified by Micco *et al.* [15]. Fifty ml of each milk sample were mixed with 70 mL of methanol-acetone (5:2 v/v) solution in the presence of 7.2% sodium bicarbonate, and extracted according to the method of Domgang *et al.* [8]. Urine and serum samples were extracted using the methods of Tongtavuch and Chutima [16] and Wray and Hayes [17], respectively.

*Aflatoxin analysis* Extracts from all the samples were analysed by the HPLC method (Beckman Model 110A) for the identification and quantification of the different types of aflatoxins (B1, B2, G1, G2, B2a and M1). The HPLC column used was a ODS-Sil-X-10 μm reverse phase column (4 × 250 mm, Perkin Elmer). Samples were injected (20 μL) through a Perisorb RP 18, 10−40 μm pre-column (4.9 × 50 mm, Waters) at 2,000 psi pressure. The mobile phase used was bidistilled water-acetonitrile-methanol (16:5:5 v/v/v), at a rate of 1 mL·min^−1^. Aflatoxins were detected using a Beckman fluorescent detector (Type 157) set at excitation wavelength of 365 nm and at emission 430 nm. For each extract, control aflatoxins were run. Their proportions were calculated using a Hewlett Packard Integrator Model 3309. AFB_1_ was converted to its derivative, AFB_2a_ using trifluoroacetic acid before analysis. Since eggs contain AFB_2a_, egg samples were extracted using two methods: that described by Truckness and Stoloff [18] which is specific for AFB_1_, and the method of Micco *et al.* [15] which isolates both AFB_2a_ and AFB_1_. AFB_2a_ content was computed as the difference in the concentrations obtained from the two methods of extraction, after consideration of recovery rates [15].

*ELISA assay of aflatoxin:* The presence of AFB_1_ and AFM_1_ was also confirmed using an immuno-enzymatic commercial kit based on monoclonal antibodies specific for each of them (Transia, Lyon France). Briefly, the extract was evaporated to dryness and taken up in the sample buffer provided in the kit; 50 μL of the sample was then pipetted into duplicate wells of an ELISA plate sensitised with the monoclonal antibody specific for aflatoxin AFB_1_ or AFM_1._ Following incubation and washing, 50 μL of the specific monoclonal antibody conjugated to horseradish peroxidase dissolved in the conjugate buffer provided in the kit was added, incubated and washed again before the substrate was added and incubated for colour development as recommended by the suppliers. Colour intensity was read off an ELISA reader provided with the kit. Standards included in the kit allowed the calculation of aflatoxin in the extract assayed.

*Detection of HbsAg* The Hepatitis B virus surface antigen was detected in serum samples using a sandwich method according to the procedure described in the commercial kit (Behring, Marburg, Germany)

### 2.3. Statistical Analysis

Values were expressed as means or frequencies. Data obtained in this study were analysed using ANOVA or Dunnett tests. The frequency of contamination was analysed using the Chi-square test. The level of significance was set at p < 0.05.

## 3. Results

### 3.1. Aflatoxin Content in Egg Samples

**Figure 1.**
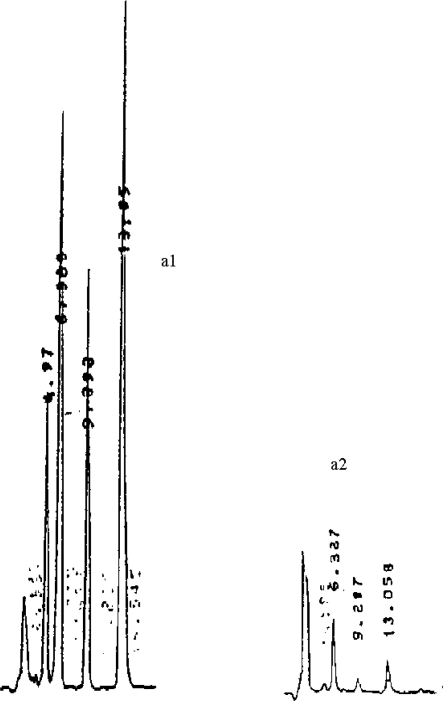

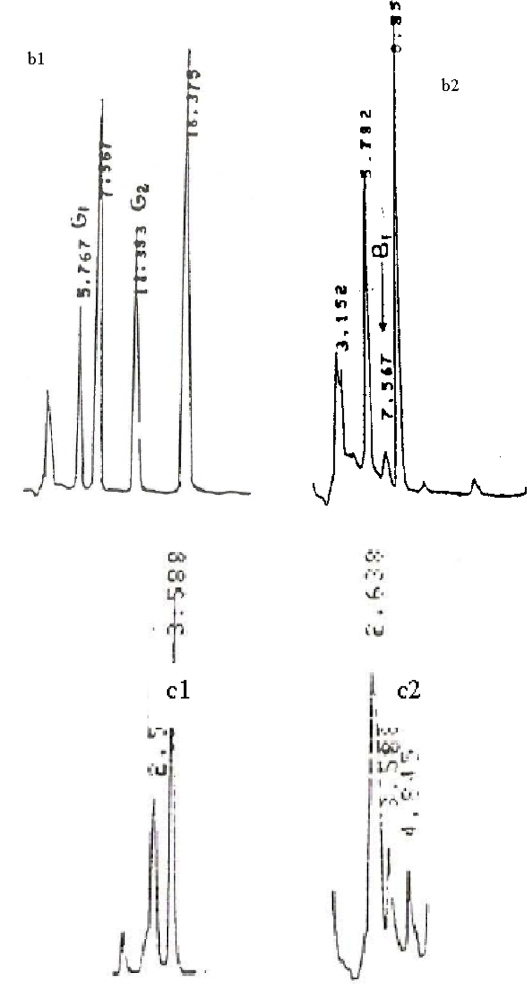
HPLC analysis of Aflatoxins in egg extracted using both methods. (a1): Aflatoxins G_1_, B_1_, B_2_, G_2_ standards with R_T_ 4.97, 6.325, 9.929 and 13.05 min, respectively. (a2): Extract using procedure [14]; R_T_ 6.327, 9.287 and 13.058 min for AFB_1_+AFB_2a_, AFG_2_ and AFB_2_, respectively. Concentrations are 0.154, 0.074 and 0.051 ppb for respectively AFB_1_+AFB_2a_, AFG_2_ and AFB_2_. (b1) Aflatoxins G_1_, B_1_, B_2_, G_2_ standards with R_T_ 5.767, 7.567, 11.353 and 16.375 min, respectively. (b2) Extract using procedure [17] R_T_ 5.782 and 7.567 min for AFG_1_ and AFB_1_ respectively. Concentrations are 0.099 and 0.390 ppb, for AFB_1_ and AFG_1_ respectively. AFB_2a_ from (a) – AFB_2a_ from (b) = 0.055 ppb according to [14]. (c1) Aflatoxin M_1_ standard with retention times 3.588 min. (c2) extract for AFM1 detection R_T_ 3.588 min.

For each type of sample, known amounts of aflatoxin were added and extracted by the various methods to determine the percentages of recovery. These varied between 87 and 96% for egg samples and urine. Figure 1 shows an HPLC chromatogram of extracts of egg samples obtained according to the procedures described in the Methods. Extraction using the method of Micco *et al*. [15] permitted the isolation of AFB_2a_ (Figure 1. a2). The presence of AFB_1_ in egg extracts was confirmed using specific monoclonal antibody-based ELISA assay. Contamination in egg samples according to various climatic regions of Cameroon varied from 25 to 52.5% with the Forest and Littoral regions having the highest levels (Table 1); toxins found were AFB_1_, AFB_2a_, AFB_2_, AFG_1_ and AFM_1_.

### 3.2. Aflatoxin in Milk

Aflatoxin M_1_ was analysed in duplicate by ELISA; 15.9% of cow milk samples had AFM_1_ at levels varying from 0.006 to 0.525 μg·L^−1^, while 3 of the 62 (4.8%) of breastfeeding milk were also positive (Table 2). Some samples showed 10 times higher values than the recommended maximum (Table 2).

### 3.3. AFB_1_ in Urine Samples of Malnourished Children

Urine samples from 42 children of ages ranging from 13 months to 12 years who were hospitalised in Yaounde (forest region) for malnutrition were analysed. Concomitantly, a group of 36 healthy children within the same age range and from the same area of residence were used as controls for the analysis (Table 3). A total of 35.5% of the samples from kwashiorkor children and 45.5% from the marasmic kwashiorkor children contained AFB_1_ at detectable levels (Table 4). Only 11% of control children were AFB_1_ positive (P < 0.05 Dunnett test).

### 3.4. AFB_1_ in and HbsAg in Primary Liver Cancer Patients

**Figure 2.**
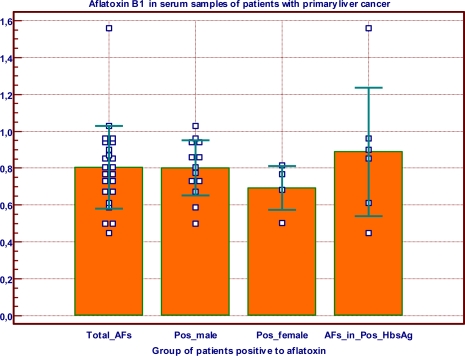
AFB_1_ content μg·L^−1^ in serum of primary liver cancer patients from Yaounde Hospital. Data were collected during a 4-year survey. Values are presented as Mean ± SD. Total _AFs (total patients positive for AF); Pos_male (male patients positive for AF); Pos_female (female patients positive for AF); AFs_in_Pos_HbsAg (patients positive for both HbsAg and AF). There was no significant differences in the serum content of AFB1 in the different groups (P > 0.05 ANOVA).

Sera of liver cancer patients of age ranging between 10 and 80 years were analysed for AFB_1_ and HbsAg (Table 5). Of the 36 sera, 63.9% were positive for AFB_1_ with concentrations varying from 0.450 to 1.560 μg·L^−1^, whereas 69.4% were positive for the HbsAg test; 36.1% were positive for both tests and only one patient was negative for both tests. Mean values of AFB_1_ concentrations in serum, by sex and positivity for HbsAg are shown in Figure 2. There was no significant difference in AFB_1_ concentrations with respect to sex or HbsAg status.

## 4. Discussion

Aflatoxin contamination in various foods is a major threat to the health of exposed people. We detected AFB_1_, AFB_2a_ and AFM_1_ in egg samples, thus confirming our earlier reports [7,8]. Apart from AFB_2a_ which is known to have low toxicity, the two other compounds are highly toxic [19]. This emphasizes the importance of monitoring AFB_1_ and its metabolites in poultry products. Overall, the forest region had the highest toxin contamination. Factors such as time of exposure of the hen, rate of formation of metabolites and their rate of transfer to the egg may influence egg contamination, but these factors were not evaluated during this study. It has been reported that contamination of eggs may influence the performance of chick after hatching [20]. Apart from eggs, AFB_1_ has also been detected in gizzard and chicken muscle [21].

AFM_1_ was also detected in 15.9% of the cow milk samples at levels higher than the recommended maximum (Table 2). This was probably due to the contamination of feed given to the cattle, since it is well known that AFM_1_ is usually excreted in milk two days after ingestion of feed contaminated by AFB1 [22]. In addition to the contamination of local milk, contamination has also been found with milk powder and pasteurised milk in various European countries [10,23–25].

Milk from breastfeeding mothers (4.8%) was also contaminated with AFM_1_. This implies that children might be regularly exposed to AFM_1_ via breast milk or imported processed milk from European countries. Further, AFB_1_ has been reported in cord blood of children in Nigeria and Ghana [26], meaning that it crosses the placental barrier from the mother to the foetus. *In utero* exposure of the foetus to toxins, exposure of children to environmental toxins, and the nutritional status of the infant, are considered critical for growth and risk of disease in early life. Indeed, malnutrition is one of the common problems in developing countries.

The presence of AFB_1_ in body fluids of children is a potential risk of impairment of growth during their development, since it is well known that it inhibits protein synthesis [27]. We detected AFB1 in the urine of malnourished children and there seemed to be a link between malnutrition and the presence of AFB_1_ in the urine of malnourished children (P < 0.05 Dunnett; Table 4). It is possible that the presence of the toxin is a reflection of food habits of the mothers whose knowledge, attitudes and practices with respect to food may vary considerably.

Indeed, research on humans in Benin and Togo showed a dose-response relationship between exposure to aflatoxin, degree of stunting and underweight in children under five years of age; the study population was exposed to aflatoxin, with 99% of the children having serum aflatoxin-albumin adducts [28]. Further, many researchers in African countries have reported a strong association between malnutrition and the presence aflatoxin in body fluids from children [2], although studies with periurban children and children hospitalised for kwashiokor in South Africa did not find any aflatoxin contamination [29]. As a consequence of the known effect of aflatoxin on protein synthesis, aflatoxin in body fluids of children could be a modulating factor on the rate of recovery from protein malnutrition, although it has not been shown to be responsible for the development of the condition. Further, it has been suggested that aflatoxin reduces the nutritional capacity of food by interfering with metabolic processes [30]. Species differences in the acute and chronic toxicity of AFB1 have been documented in animal, with females showing greater resistance to the toxin [31]. Our results seem to show a greater proportion (10/21 males and 6/21 females) of urine samples from male children contaminated more than samples from female children with kwashiorkor and marasmic kwashiorkor (Table 4), Since we did not follow the daily intake of aflatoxins in food consumed by male and female children, it cannot be concluded that our results corroborate the observations made in animals. Therefore, sex differences related to aflatoxin toxicity in human is still to be proven.

Exposure to AFB_1_ from early age could be a predisposing factor for primary liver cancer. Indeed, it is well known that aflatoxins, viral hepatitis, alcohol, and cigarette are high risk factors for cancer development [5]. Our results show that 63.9% of samples from primary liver cancer patients were positive for AFB_1_ while 69.4% were positive for HbsAg. We did not find any linkage between contamination with AFB_1_ and infection with HBV, but this is not surprising because the two factors are independently introduced into the subject. However, since both aflatoxin and HBV have an effect on the liver, there is need to investigate the effect of the food contamination reported here on the evolution of HBV pathology, especially the possible additive or synergistic effects, and the possible contribution of other environmental factors [32].

In conclusion, aflatoxins seem to be present in foods recommended for children like eggs and milk, at levels above recommended thresholds. A high prevalence of contamination was found in the urine and blood of malnourished children with kwashiorkor and marasmic kwashiorkor; people with primary liver cancer also had a high prevalence of contamination with aflatoxin and infection with HBV. It is necessary to investigate if aflatoxin has an additive or synergistic effect on the pathology of HBV.

## Figures and Tables

**Table 1.** Frequency of contamination and aflatoxin content in egg sample (ppb) according to climatic areas.

**Climatic areas**	**Samples**		**Type of AFs in sample**					
	**Analysed**	**Positive^(..)^**	**B_1_**	**B_2_**	**B_2a_**	**G_1_**	**M_1_**	**Range of AFs content in ppb**
Forest	23	12 (52.5)	1	1	10	4	2	0.021–7.604
Savannah and steppe	8	2 (25)	0	-	0	-	2	0.003–0.008
Littoral	12	6 (50)	0	-	5	1	1	0.126–0.768
Mountain	19	8 (42.1)	3		2	1	5	0.002–7.200

Total	62	28 (45.2)	4	1	17	6	10	0,820±1,707 *

( ) percentage of positive sample; values are presented as aflatoxin content for each type of aflatoxin found by HPLC. Each sample was analysed three times to give an estimate of the contamination;

* mean value ± SD.

**Table 2.** Aflatoxin M_1_ contamination of milk.

**Type of milk**	**Sample analysed**	**Positive Samples**	**AFM1 content (μg/L)**	Sample with level < 0.05* μg·L^−1^	**Sample with level > 0.05 μg·L^−1^**
Cow milk	63	10 (15.9%)	0.006 to 0.527	4	6
Breastfeeding milk	62	3 (4.8%)	0.005 to 0.625	2	1

* Maximum tolerated level from the FAO in 2003 [10].

**Table 3.** Age and sex distribution of control and malnourished children.

**Ages (Years) Groups**	**Gender**	**1–3**	**4–5**	**6–9**	**10–12**	**Total**
Kwashiorkor	Male	5	3	3	0	11
	Female	3	1	5	2	11
Marasmic Kwashiorkor	Male	4	3	1	1	9
	Female	3	2	4	2	11
Control	Male	2	4	7	7	20
	Female	2	2	6	6	16

**Table 4.** Aflatoxin B_1_ content in urines of malnourished children.

**Children Groups**	**Male**		**female**		**Total**		**AFB1 (μg·L^−1^)**

	**Total**	**Positive**	**Total**	**Positive**	**Total**	**Positive**	
Kwashiorkor	16	7 (43.8%)	15	4 (26.6%)	31	11 (35.5)	0.109–2.840*
Marasmic kwashiorkor	5	3 (60%)	6	2 (33.3%)	11	5 (45.5%)	0.109–0.864*
Control	20	3 (15%)	16	1 (6.3%)	36	4 (11.1%)	0.07–0.155

Data from 24-hour urine collected from children admitted at the University Teaching Hospital in Yaounde and suffering from kwashiorkor or marasmic kwashiorkor, and from 36 healthy children within the same age range and area of residence,

* Statistically significant difference compared to the control group at P < 0.05 Dunnett test.

**Table 5.** AFB_1_ and HbsAg in sera of primary liver cancer patients from Yaounde hospital.

**Age Range in years**	**Total number of patients**	**Positive patients to HbsAg**		**Positive patients to AFB1**		**Positive patients for both AFB1 and HbsAg**	

		**Number**		**Number**		**Number**	

		**Male**	**Female**	**Male**	**Female**	**Male**	**Female**
10*–*20	1	1	-	1	-	1	-
20*–*30	4	2	1	2	1	2	-
30*–*40	11	6	1	5	3	4	-
40*–*50	6	4	1	3	-	2	-
50*–*60	7	6	-	4	-	3	-
60*–*70	4	2	-	2	-	1	-
70*–*80	3	1	-	2	-	-	-

Total	36	22(61.1%)	3(8.3%)	19(52.7%)	4(11.1%)	13(36.1%)	-

Data collected during a four year survey (1991−1995). Blood samples were also collected from cancer patients admitted in two reference hospitals in Yaounde and who accepted to participate in the study by giving their informed consent. ( ) Total percentage of serum samples positive to AFB_1_ or HbsAg.
